# Analysis of factors influencing the outpatient workload at Chinese health centres

**DOI:** 10.1186/1472-6963-10-151

**Published:** 2010-06-05

**Authors:** Jing Xu, Wenxin Wang, Yongbin Li, Juan Zhang, Milena Pavlova, Hua Liu, Ping Yin, Zuxun Lu

**Affiliations:** 1 Department of Social Medicine and Health Management, School of Public Health, Tongji Medical College, Huazhong University of Science & Technology, 13 Hangkong Road, Wuhan, Hubei Province, 430030, PR China; 2 Department of Health Organisation, Policy and Economics, Faculty of Health, Medicine and Life Sciences, CAPHRI, Maastricht University Medical Centre, Maastricht University, The Netherlands; 3 Department of Child Health Care of Wuhan City Maternal and Child Health Care Center, 100 Xianggang Road, Wuhan, Hubei Province, 430030, PR China; 4 Department of Epidemiology and Health Statistics, School of Public Health in Tongji Medical College, Huazhong University of Science & Technology, 13 Hangkong Road, Wuhan, Hubei Province, 430030, PR China

## Abstract

**Background:**

Although the community health service system is now established in China, the utilisation of the community health service institutions is low due to the lack of a gate-keeping role of the primary health service providers and referrals among the three-tiered health service institutions. In addition to this, patients who can afford to pay, often seek best services in big hospitals to guarantee the quality of care. Thus, the need of guiding the patients to the community health services and increasing the utilisation of the community health service institutions is becoming an urgent problem, which hinders the future development of community health services. This study focuses on the question of how to increase the utilisation of Chinese community health centres (HCs).

**Methods:**

A cross-sectional Base-line Survey of Chinese City Community Health Service System Building using the multi-staged cluster sampling was conducted to collect data from all HCs in 28 key contact cities. Relevant indicators of totally 1790 HCs were analysed. The statistical methods included ANONVA and logistic regression.

**Results and Conclusions:**

The analysis suggested several key factors for increasing the outpatient workload (OW) at the HCs: establishing an adequate referral system among the different levels of the health system; enhancing the qualification of health personnel and increasing the compensation by the health insurance for services provided at HCs. Other key factors with a positive effect on the OW included: the government ownership of the HCs, the scale of the institutions, the medical equipment used, the mix of health services provided, and the women in childbearing age in the residence.

## Background

In order to enhance the efficiency in the Chinese health system, a market-oriented reform under the government's planning was introduced in the health sector in 1980s. The two of the main measures adopted by the government to establish a competitive health service market, included the introduction of a competition among the three-tiered health service institutions and the introduction of patients' out-of-pocket payment directly to the health service providers. In this new health service market, there is a price system laid down by the government for services provided by the health service institutions at different levels. The health service institutions can compete with each other to attract patients from the same level or from a different level, while the patients can freely choose the health service providers they prefer most, and pay the chosen provider directly out-of-pocket. Thus, the traditional gate-keeping role of the primary health service institutions is deserted.

The health service institutions compete for patients to be able to generate the revenue that they require. The competition stimulates the health service institutions to update their facilities and equipment, enlarge their scale, enrol excellent physicians by offering liberal wages and benefits, and adopt a positive attitude towards patients' preferences. Thus, there is no waiting list and the efficiency of health reform is overall achieved. But simultaneously, there is a concentration of superior health resources and patients at the third-tiered health service institutions as well as a decline in the use of primary and secondary health service institutions. This results in a serious equity problem, especially with regard to patients without high income, who cannot afford to pay directly to physicians. Therefore, the Chinese government endeavoured to develop a convenient and affordable primary health services - community health service - for the city residents. This mainly involved a transformation of the former health service institutions at the primary level and some health service institutions at the secondary level at the end of 1990s [1].

After a decade of preparations and developments, the Chinese community health service system has been preliminarily established. Thus, a health centre (HC) is set up on every street in the cities included in the reform. A typical HC covers from 30,000 to 50,000 residents and it is equipped with up to 50 inpatient beds. At the same time, a health station (HS) is set up in every resident housing estate, which covers 3000 residents and it is not equipped with inpatient bed. The community health service institutions, including both HCs and HSs, provide six types of community health services: disease prevention, treatment, care, rehabilitation, health education and family planning. Most of the HCs and HSs are owned by the government while the rest are owned by the enterprises or social organisations, and individuals. Most of them are autonomous in its management [2-4].

**Figure 1:**
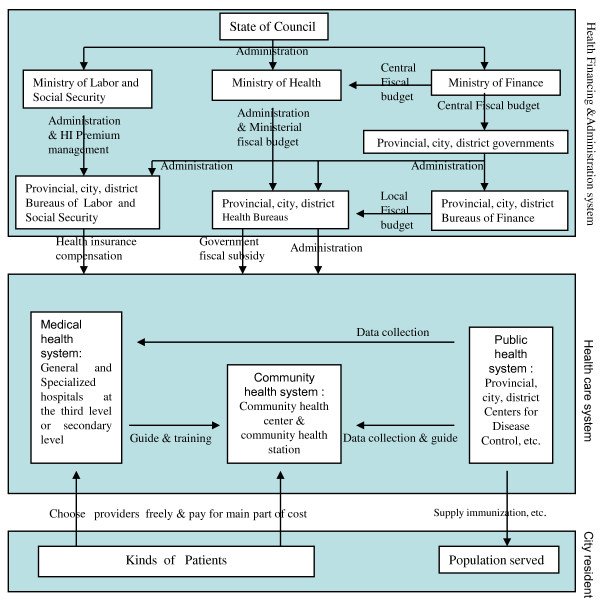
**Chinese city health system (source: by the authors)**.

There are three main mechanisms of financing the HCs and HSs (See Figure 1). The fiscal subsidy from the government is mainly a compensation for the staff basic salaries and facilities, while the health insurance (HI) compensates a proportion of other medical costs. The main part of the income of HCs and HSs is paid by the patients. Hitherto, the city health insurance system (including the Urban Worker Basic Medical Insurance and Urban Resident Basic Medical Insurance under the charge of the Ministry of Labour and Social Security) has not been fully set up due to various unsolved technical issues. At present, it mainly compensates the medical costs of the third-tiered health service institutions. Therefore, only some HCs and HSs obtained health insurance compensations (HICs)[5-7].

Although the community health service system is now established, the utilisation of the community health service institutions is low due to the lack of a gate-keeping role of the primary health service providers and due to referrals among the three-tiered health service institutions. In addition to this, patients who can afford to pay, often seek best services in big hospitals to guarantee the quality of care. Thus, the need of guiding the patients to the community health services and increasing the utilisation of the community health service institutions is becoming an urgent problem, which hinders the future development of community health services. This problem can be explicated by the exploration of the outpatient workload (OW) in the Chinese health system. The data from the Base-line Survey of HCs in 28 Chinese cities in 2007 showed that there were 40,000 residents served and 40,000 average annual outpatient visits in 2006[8,9]. These levels were very low compared to the population size in these cities, and specifically to the annual number of outpatient service users in China. This study focuses on the question of how to increase the utilisation of Chinese HCs.

Chinese academic journals offer several articles on the analysis of factors that influence the OW of hospitals from the patient's point of view [10-13]. However, there is a lack of papers on how to influence the OW of Chinese HCs. This gap in research is observed in both international journals and domestic journals. Moreover, in China, the coordination among the competing health service institutions at different levels becomes a concern. Thus, the need to guide the patients to seek health services starting from the HCs is also a relevant policy issue.

According to Josep et al. (2004)[14], it is necessary to understand the problems in a health system by undertaking an overall "health system perspective". Thus, the low utilisation of Chinese community HCs should not be examined by considering only the traditional view of patients. It needs to be understood by the examination of the health system as a whole. Currently, most of the Chinese health service institutions belong to the government. Therefore, the health system management and health service provision are combined together (See Figure 1). This requires a special attention when analysing health system problems, such as the low OW of Chinese HCs. The Baseline Survey of The National City Community Health Service System Building Project was designed to investigate the performance of HCs/HSs from the point of view of health service provision and management. Based on the Baseline Survey, in this paper, the factors that influence the OW of Chinese HCs were analysed from the perspective of health service provision and management. Thus, the aim of the paper is to identify the main factors that could help to increase the OW of Chinese HCs, and to provide strategies for the adjustment of national health policy in developing the community health service. Moreover, the need of communicating the Chinese experience and providing lessons for other countries with a similar problem is also a motivation for preparing this paper.

## Methods

### Data source and sampling

The data for this paper were extracted from the database set up by the Baseline Survey of The National City Community Health Service System Building Project carried out in 28 cities during the period August - December 2007. All 28 cities, including 12 cities in the east, 8 cities in the middle and 8 cities in the west of China, were selected by a multi-staged cluster sampling method in terms of economic and politic characteristics, city size and the level of development of community health services. In 2007, totally 3125 HCs were set up in the country, while all HCs in the 28 cities (totally 1917 HCs) were investigated in the Baseline Survey. Thus, these HCs served as a representative sample of the overall development of community health services in urban China [2,3,15].

The data were collected using a uniform questionnaire. The questionnaire was developed in several steps: (1) firstly, a pool of indicators was set up (based on foreign and domestic literature reviews) including all indicators that reflect relevant information on Chinese community health service; (2) secondly, the indicator pool was screened using a Delphi technique, and the indicators that conformed to the aim of the project were included in the questionnaire; (3) finally, the questionnaire was pretested before it was used. The questionnaire mainly included questions on the district demographic characteristics, characteristics of the basic setup of the HCs, health personnel, health staff training, HCs income and expense, and the health service provided. In this paper, only relevant indicators for the HCs from the Baseline Survey Questionnaire were selected for the analysis.

### Project quality control

The Baseline Survey was conducted by the MOH using the uniform questionnaire. Initially, the survey and questionnaire were designed by the project team. A training program (under the organisation of the MOH) was conducted for all investigators in the 28 key contact cities. Secondly, the local health bureau in every key contact city conducted the investigation under the supervision of MOH. The MOH enjoined the city health bureaus to collect the data of all HCs under their jurisdictions. Before sending the questionnaires to all community HCs under the jurisdiction, the local bureaus trained the persons who were responsible for filling in the questionnaire. Then, these persons filled in the questionnaire for their HCs and sent the data to local health bureaus. Finally, the field data were inserted into the database designed by the project team (twice independently), and were uploaded by the respective local health bureaus. The screening of the dataset was carried out by the team members.

### Variables and data analysis

A set of 36 indicators (potentially influencing) the OW of HCs have been chosen for this analysis and were divided into four categories: 8 indicators for policy and management, 4 indicators for financing, 11 indicators for continuum of care, and 13 indicators for quality of care. (See Table 1 and Table 2).

**Table 1:** The results of ANOVA of the categorical variables for the two groups of HCs

	LOWG		HOWG		Total			
Categorical variable	Yes	N*(%)	Yes	N* (%)	Yes	N* (%)	*x*^2^	p
**1. Policy and Management**								
1.1 Whether HC is a unit of corporate sole	890	(71.95)	269	(48.64)	1159	(64.75)	90.9316	<0001
1.2 Whether separate management of income and expenditure in two lines	430	(34.76)	154	(27.85)	584	(32.63)	8.3095	0.0039
1.3 Whether all-staff employment management	1009	(81.57)	465	(84.09)	1474	(82.35)	1.6674	0.1966

1.4 Ownership of HC (total)	1237	(100)	553	(100)	1790	(100)		
Government-owned HC	915	(73.97)	466	(84.27)	1381	(77.15)	24.2437	<0001
Enterprise-owned HC	235	(19.00)	59	(10.67)	294	(16.42)		
Private HC	55	(4.45)	20	(3.62)	75	(4.19)		
Other holder-owned HC	32	(2.59)	8	(1.45)	40	(2.23)		

1.5 HC designated by health insurance (total)	1237	(100)	553	(100)	1790	(100)		
Not designated by health insurance	553	(44.7)	164	(29.66)	717	(40.06)	123.7698	<0001
Outpatient designated	348	(28.13)	302	(54.61)	650	(36.31)		
Inpatient designated	38	(3.07)	21	(3.8)	59	(3.30)		
Both outpatient and inpatient designated	298	(24.09)	66	(11.93)	364	(20.34)		

1.6 Whether HC owns the house (total)	1237	(100)	553	(100)	1790	(100)		
House is used for free	344	(27.81)	184	(33.27)	528	(29.5)	40.499	<0001
House is owned by HC	538	(43.49)	154	(27.85)	692	692 (38.66)		
House is rented by HC	355	355 (28.7)	215	(38.88)	570	(31.84)		

1.7 How HC pays for the rented house (total)	892	(100)	369	(100)	1262	(100)		
Rent subsidised by the government	223	(24.97)	89	(24.12)	312	(24.72)	12.3449	0.0063
Rent partly subsidised by the government	144	(16.13)	41	(11.11)	185	(14.66)		
Rent self-raised	432	(48.38)	213	(57.72)	645	645 (51.11)		
Rent paid by other sources	94	(10.53)	26	(7.05)	120	120 (9.51)		

**4. Quality of care**								
**4.1 Facilities**								
4.1.1 HC with Chinese medicine department	1008	(81.49)	396	(71.61)	1404	(78.44)	22.0464	<0001
4.1.2 HC with Chinese medicine dispensary	882	882 (71.3)	337	(60.94)	1219	1219 (68.1)	18.8855	<0001

4.1.3 HC with medical equipment ^#^								
With rehabilitation physiotherapy facilities	932	(75.34)	453	(81.92)	1385	(77.37)	9.432	0.0021
With electrocardiogram machine	1174	(94.91)	534	(96.56)	1708	(95.42)	2.4009	0.1213
With blood sugar instrument	1144	(92.48)	519	(93.85)	1663	(92.91)	1.088	0.2969
With biochemistry analysis instrument	930	(75.18)	399	(72.15)	1329	(74.25)	1.8349	0.1756
With B ultrasound instrument	1052	(85.04)	456	(82.46)	1508	(84.25)	1.9243	0.1654
With centrifugal machine	849	(68.63)	356	(64.38)	1205	(67.32)	3.1488	0.076
With X ray machine	879	(71.06)	290	(52.44)	1169	1169 (65.31)	58.4652	<0001

Note: * N indicates the number of HCs in the groups LOWG or HOWG, which responded with "yes" for a given indicator.

^# ^Every HC does not necessarily own these seven medical equipments, maybe it only owns some of them.

**Table 2:** The results of ANOVA of the numerical variables for the two groups of the HCs

	HOWG	HOWG		
Numerical variable	Mean ± Std	Mean ± Std	t	p
**1. Policy and Management**				
1.1 Management between HC and HS				
HS owned by HC	1.83 ± 3.24	1.68 ± 3.44	0.92	0.3597
HS not owned by HC but receiving HC guide	1.18 ± 4.75	0.54 ± 2.55	3.72	0.0002
**2. Financing**				
2.1 Percentage of treatment income in total income (%)	22.11 ± 17.78	26.00 ± 17.29	-4.32	<0001
2.2 Percentage of drug income in total income (%)	39.47 ± 21.76	39.17 ± 20.00	0.29	0.7745
2.3 Percentage of fiscal income in total income (%)	23.42 ± 24.57	18.43 ± 18.43	4.75	<0001
2.4 Percentage of health insurance in total income (%)	9.46 ± 14.36	12.50 ± 14.73	-4.1	<0001
**3. Continuum of care**				
**3.1 Demographic characteristics of population served**				
3.1.1 Percentage of the population above 60 in total population (%)	11.09 ± 7.83	7.80 ± 7.55	8.31	<0001
3.1.2 Percentage of children aged in 0 to 6 in total population (%)	3.85 ± 3.30	4.20 ± 4.11	-1.75	0.0807
3.1.3 Percentage of baby aged in 0 to 1 in total population (%)	0.78 ± 1.06	0.97 ± 1.27	-3.09	0.0021
3.1.4 Percentage of pregnant women in total population (%)	0.71 ± 1.79	0.60 ± 0.79	1.79	0.0743
3.1.5 Percentage of alive newborn baby in total population (%)	0.61 ± 1.38	0.54 ± 0.57	1.54	0.1242
3.1.6 Percentage of women in childbearing age in total population (%)	21.48 ± 13.45	27.96 ± 19.15	-7.18	<0001
3.1.7 Percentage of mental patient in total population (%)	0.21 ± 1.03	0.15 ± 0.40	1.74	0.0824
3.1.8 Percentage of disabled patient in total population (%)	1.10 ± 1.54	0.66 ± 1.04	7.18	<0001
3.1.9 Percentage of people with pension in total population (%)	1.92 ± 2.81	1.05 ± 2.54	6.49	<0001
**3.2 Referral**				
3.2.1 Patients referred to the upper level institutions	132.68 ± 591.09	445.68 ± 1742.78	-4.12	<0001
3.2.2 Patients referred to the lower level institutions	40.38 ± 215.17	123.29 ± 577.65	-3.28	0.0011
**4. Quality of care**				
**4.1 Facilities**				
4.1.1 Fixed asset owned by the HC (10,000 yuan)	433.86 ± 879.91	342.02 ± 776.69	2.22	0.0268
4.1.2 Area of HC house ( M^2^)	2176.22 ± 2733.49	1647.48 ± 1941.14	4.66	<0001
4.1.3 Drug kinds				
West medicine	359.68 ± 296.29	392.08 ± 562.53	-1.28	0.2018
Chinese patent medicine	148.31 ± 126.51	157.05 ± 273.01	-0.72	0.4723
4.1.4 Beds				
Current inpatient bed	28.94 ± 44.47	15.59 ± 29.40	7.51	<0001
Current observation bed	0.52 ± 1.12	0.51 ± 1.00	0.21	0.8349
**4.2 Human resources**				
4.2.1 Total member of current staff	56.49 ± 93.93	42.33 ± 49.87	4.15	<0001
Temporary member of staff`	12.41 ± 18.39	14.90 ± 18.60	-2.64	0.0084
4.2.2 Total member of health technicians	43.84 ± 44.68	32.56 ± 35.84	5.68	<0001
Clinical doctor	19.44 ± 20.77	13.07 ± 14.53	7.43	<0001
Dentist	1.12 ± 1.86	0.99 ± 1.80	1.43	0.1519
4.2.3 Physician with education certificate	20.70 ± 24.08	15.25 ± 16.69	5.53	<0001
Physician with postgraduate certificate	0.29 ± 1.36	0.35 ± 1.59	-0.71	0.4781
Physician with undergraduate certificate	6.68 ± 11.72	5.05 ± 7.55	3.53	0.0004
Physician with 3-year education certificate	8.05 ± 9.50	5.88 ± 6.66	5.55	<0001
Physician with 2-year education certificate	4.68 ± 7.03	3.38 ± 4.86	4.53	<0001
Physician with less than two year education certificate	0.98 ± 3.51	0.59 ± 1.94	3.06	0.0023
4.2.4 New physician enrolled into in 3 year	2.94 ± 5.88	3.00 ± 4.09	-0.25	0.8017
New physician with postgraduate certificate	0.08 ± 0.66	0.09 ± 0.38	-0.58	0.5635
New physician with undergraduate certificate	1.45 ± 4.08	1.22 ± 2.14	1.57	0.1172
New physician with 3-year education certificate	1.05 ± 2.20	1.25 ± 2.21	-1.77	0.0774
New physician with 2-year education certificate	0.32 ± 1.18	0.40 ± 1.07	-1.33	0.1835
New physician less than 2 years education certificate	0.05 ± 0.88	0.05 ± 0.42	-0.09	0.9289
4.2.5 Nurse with education certificate	13.44 ± 16.88	9.68 ± 11.32	5.53	<0001
Nurse with postgraduate certificate	0.02 ± 0.40	0.00 ± 0.06	1.41	0.1595
Nurse with undergraduate certificate	0.48 ± 1.41	0.46 ± 1.90	0.22	0.8238
Nurse with 3-year education certificate	4.80 ± 7.58	3.24 ± 5.18	5.08	<0001
Nurse with 2-year education certificate	7.36 ± 9.57	5.65 ± 6.46	4.44	<0001
Nurse with less than 2 years education certificate	0.78 ± 2.59	0.33 ± 1.17	4.99	<0001
4.2.6 Family medicine and community nursing training				
Physician receiving family medicine guideline training	1.70 ± 5.66	1.54 ± 4.75	0.63	0.5281
Physician receiving the family medicine training	8.04 ± 9.87	7.68 ± 9.74	0.71	0.4749
Nurse receiving the community nursing training	6.20 ± 9.19	5.79 ± 7.87	0.96	0.3364
Staff passing the national middle technical qualification examination	1.18 ± 4.17	1.21 ± 3.82	-0.14	0.8857

In total, 1917 HCs were investigated, of which 1790 HCs reported the annual outpatient visits. The purpose of the analysis was to find out why some HCs had a higher OW while others had a lower OW. Therefore, the sample of 1790 HCs was divided into two groups: HCs with high OW (HOWG) and HCs with low OW (LOWG). There is no standard to decide whether a HC has a high or low OW. Therefore, we decided to take the average annual outpatient visits per clinical doctor of all 1790 HCs as a criterion. Then, we compared the average annual outpatient visits per clinical doctor for every HC to this average. The HCs whose average annual outpatient visits per clinical doctor were equal or higher than the average, were included in the group HOWG (553 HCs), otherwise, they were included in the group LOWG (1237 HCs).

To analyse the factors that influence the OW of Chinese HCs, the study adopted the method of statistic analysis provided in other recent studies on similar topics [11-14]. The comparison between the two groups was done by ANOVA, and then, the influence of indicators on the OW was analysed by logistic regression.

Thus, the dependent variable in logistic regression was the average annual outpatient visits per clinical doctor while the independent variables were the 36 indicators explained above but divided into 10 categorical indicators (Table 1) and 26 numerical indicators (Table 2). The goodness of fit of the logistic regression models appeared adequate.

## Results

### The comparison between the two groups of HCs for categorical indicators

As indicated in Table 1, there was a significant difference between the two groups of HCs (HOWG and LOWH) for all categorical indicators except for "whether all-staff employment management" in policy and management, and "electrocardiogram machine, blood sugar instrument, biochemistry analysis instrument, B ultrasound instrument, centrifugal machine" in quality of care. Among the categorical indicators with a significant difference between the two groups of HCs, the mean values for HOWG were higher than these for LOWG in case of "government-owned HC, outpatient designated, inpatient designated, house is used for free, house is rented by HC, and rent is raised by HC itself". For other indicators, the mean values for HOWG were lower than those for LOWG.

### The comparison between the two groups of HCs in numerical indicators

Table 2 suggests a significant difference between the two groups of HCs for all numerical indicators except for "HS owned by HC" in policy and management, "percentage of drug income and total income" in financing, "percentage of children aged in 0 to 6 in total population, percentage of pregnant women in total population, percentage of mental patient in total population served" in continuum of care, and "drug kinds, current observation beds, physician with postgraduate certificate, new physician with education certificate enrolled into in 3 year, nurse with postgraduate certificate, and the family medicine and community nursing training" in quality of care.

Among the indicators with a significant difference between the two groups, the mean values for HOWG were higher than those for LOWG in "percentage of treatment income in total income, percentage of health insurance in total income" in financing, "percentage of baby aged in 0 to 1 in total population served, percentage of women in childbearing age in total population served, patients referred to the upper level institutions, patients referred to the lower level institutions" in continuum of care, and "temporary member of the staff" in quality of care. For other indicators, the mean values for HOWG were lower than those of LOWG.

### The results of the logistic regression analysis

As indicated in Table 3, in total, 22 independent variables remained in the model. The variables that were found to increase the OW of HOWG compared to LOWG included: "HC owned by the government" in policy and management, "percentage of health insurance in total income" in financing, "percentage of women in childbearing age in total population, patients referred to the upper level institutions" in continuum of care, and "rehabilitation physiotherapy facilities, biochemistry analysis instrument, total member of current staff, temporary member of the staff, clinical doctor, dentist, new physician with postgraduate certificate, nurse receiving the community nursing training" in quality of care.

**Table 3:** The results of multiple factor logistic regression analysis

Variables	Estimate (*b*)	Standard Error	Chi-Square	Pr > ChiSq	OR	95% Wald CL (LL-HL)	
Intercept	-1.5183	0.2507	36.6908	<0001			
Patient referred to the upper level institutions	**0.7116**	0.1522	21.8706	<0001	**2.037**	1.512	2.745
HC is owned by the government	**0.7001**	0.1608	18.9623	<0001	**2.014**	1.47	2.76
New physician with postgraduate certificate	**0.6541**	0.2579	6.4339	0.0112	**1.923**	1.16	3.189
Total member of current staff	**0.4558**	0.2226	4.1908	0.0406	**1.577**	1.02	2.44
Nurse receiving the community nursing training	**0.4447**	0.1866	5.6811	0.0171	**1.56**	1.082	2.249
Biochemistry analysis instrument	**0.4384**	0.1571	7.7855	0.0053	**1.55**	1.139	2.109
Dentist	**0.3835**	0.1632	5.5232	0.0188	**1.467**	1.066	2.02
Temporary member of staff	**0.3596**	0.1451	6.1453	0.0132	**1.433**	1.078	1.904
Percentage of health insurance in total income	**0.3162**	0.1588	3.9653	0.0464	**1.372**	1.005	1.873
Rehabilitation physiotherapy facilities	**0.3115**	0.1538	4.1023	0.0428	**1.365**	1.01	1.846
Percentage of women in childbearing age in total population served	**0.2457**	0.1245	3.8933	0.0485	**1.278**	1.002	1.632
Physician with 2-year education certificate	-0.2372	0.1579	2.2569	0.133	0.789	0.579	1.075
Percentage of pregnant women in total population served	-0.2762	0.1356	4.1501	0.0416	0.759	0.582	0.99
House is owned by HC	-0.2815	0.1302	4.6745	0.0306	0.755	0.585	0.974
HS not owned by HC, but receiving HC guide	-0.3375	0.1514	4.9696	0.0258	0.714	0.53	0.96
Nurse with 3-year education certificate	-0.3387	0.1686	4.0377	0.0445	0.713	0.512	0.992
Physician with less than two year education certificate	-0.3498	0.1588	4.8493	0.0277	0.705	0.516	0.962
HC is a unit of corporate sole	-0.4391	0.1478	8.8288	0.003	0.645	0.483	0.861
Nurse with 2-year education certificate	-0.48	0.161	8.8829	0.0029	0.619	0.451	0.848
Percentage of people with pension in total population served	-0.5643	0.144	15.3659	<0001	0.569	0.429	0.754
Percentage of fiscal income in total income	-0.6278	0.1382	20.6426	<0001	0.534	0.407	0.7
Both the outpatient and inpatient are designated	-0.6308	0.2112	8.9166	0.0028	0.532	0.352	0.805
Clinical doctors	-0.9034	0.2118	18.1933	<0001	0.405	0.268	0.614

Notes: For every numerical variable: 0 = lower than the average mean value; 1 = higher than the average mean value; For every categorical variable: 0 = no 1 = yes; CL: confident limits; LL: Low Limit; HL: High Limit; OR > 1: it means that this variable is a protective factor for the HC with high OW or a factor to increase the OW of HC with high OW comparing to the HC with low OW; OR < 1: it means that this variable is a risk factor for the HC with high OW or a factor to decrease the OW of HC with high OW comparing to the HC with low OW.

The variables that were found to decrease the OW of HOWG compared to LOWG included: "whether HC is a unit of corporate sole, both outpatient and inpatient designated, HS not owned by HC but receiving HC guide, house is owned by HC" in policy and management; "percentage of fiscal income in total income" in financing; "percentage of pregnant women in total population, and percentage of people with pension in total population" in continuum of care, and "clinical doctor number, physician with 2-year education certificate and less than 2-year education certificate, nurse with 3-year education certificate and 2-year education certificate" in quality of care.

### Relevance of the findings

The results of the analysis suggest the following implications for increasing the OW at HCs:

■ There is a need of a referral system between institutions at different levels.

In particular, "patients referred to the upper level institutions" was ranked as first, and the mean value of this indicator for HOWG was higher than that for LOWG. Moreover, there was a significant difference between the two groups of HCs in this indicator. This implied that the OW of the HCs could be increased by the continuum of care built by the referral system.

■ The government ownership of the HC is an important factor to increase the OW. Specifically, "government-owned HC" was ranked as second, and the mean value of this indicator for HOWG was higher than that for LOWG. Moreover, there was a significant difference between the two groups in this indicator. This implied that patients were more willing to use the HCs owned by the government. Thus, the ownership of the HCs could be a consideration in increasing the OW.

■ The qualification of health personnel is the key for improving the quality of care.

The indicators "new physician with postgraduate certificate" and "nurse receiving community nursing training" were ranked as third and fifth. However, there was no significant difference in the mean values of these indicators between the two groups of HCs. Nevertheless, this implied that patient trusted more the health personnel with higher education and proper professional training. Thus, it is highly important to enhance the quality of care by improving the professional qualification of the health personnel in the HCs.

■ The scale of the institution, medical equipment used, and mix of health services provided are important factors for attracting patients to the HC.

The indicator "total member of current staff, temporary member of staff, and dentist" reflected the scale of the HCs. The value implied that patients preferred to visit the large HCs, which were perceived as more reliable in medical quality. The indicator "biochemistry analysis instrument and rehabilitation physiotherapy facilities" reflected the medical equipment used and the mix of health services provided by the HCs. Their values indicated that patients preferred the HCs with good facilities and multiple services provided.

■ It is important for the HCs to have HIC.

Regarding the indicator "percentage of health insurance income in total income", the mean value for HOWG was higher than that for LOWG, and there was a significant difference between the two groups of HCs. This implied that HIC could help to attract patients to the HCs.

■ The women in childbearing age can influence the OW of the HCs.

The mean value of the indicator "percentage of women in childbearing age in total population" was higher for HOWG than for LOWG, and there was a significant difference between the two groups. This implied that the women in childbearing age would need more health services. Thus, it could influence the OW of HCs in their residence.

## Discussion

The findings reported in this paper indicated several key strategies for increasing the OW at the HCs. Each of these strategies is discussed subsequently.

### There is a need of a referral system between institutions at different levels

The present Chinese health system is in disorder. In particular, there is no regulation on the first contact of health system through primary health service institutions, and there is a lack of classification of patients regarding the different levels of health service institutions. Thus, a large quantity of patients with common diseases concentrates on the third-tiered health service institutions, which creates a serious waste of health resources [1].

Although the referral system starts to be implemented among the health service institutions, many patients do not have health insurance to cover health service costs while the out-of-pocket payments are the main way to pay for the services. Moreover, the competition among the different levels of health service institutions and the patients' free choice of health service providers make it hard for the large hospitals to be willing to refer patients to the HCs due to the competition between the health service institutions [1]. Therefore, the aim of the Chinese government when developing the community health services should be to guide the patients with common diseases to seek health services at the HCs. However, the role of the HCs cannot be played without the guarantee of a rational health system planning. Thus, there is a need of a referral system between health service institutions at different levels in order to increase the OW of the HCs.

### The government ownership of the HC is an important factor to increase the OW

The government-owned health service institutions usually have a solid establishment, and will not easily bankrupt in comparison to other health service institutions sponsored by other stakeholders (e.g. social organisation and individuals). Therefore, patients might choose the government-owned HCs, which are reliable in bearing the responsibility. Moreover, the government-owned HCs obtain more subsidies and supervision [1]. Therefore, the health service prices are more stable and this wins the trust of the patients. Thus, the ownership of the HCs could help to increase the OW in the HCs.

### The qualification of health personnel is the key for improving the quality of care

There is a large difference in the medical education of health personnel when comparing China to Western European countries and US. In Western European countries and US, all physicians must follow 8-9 years of medical education and training. But in China, due to the lack of medical staff, a large amount of medical students who were trained for 3-5 years, were allocated in the health service institutions. In the current competitive health service market, the physicians with a long standard medical education and excellent medical skills are abstracted by the upper-levels of the health system due to high salaries, benefits and good working conditions. At the same time, the medical staff in the primary health service institutions are mainly the ones who received a 3-5 years medical education (see Table 2), which cannot meet the high expectation of the patients [1,15-25].

Besides this, the Chinese medical educational system mainly aims to train medical specialists. Thus, most of the community physicians do not have a special training in community health services. It is only in the recent years that the health staff of the HCs started to receive a training in family medicine and community nursing in order to adapt to the changes in the health service function and to be able to provide adequate community health service. Therefore, the ability of HCs to provide multiple health services also needs to be enhanced [1]. The qualification of health personnel is the key to the improvement of the quality of care in order to increase the OW at the HCs.

### The scale of the institution, medical equipment used and mix of health services provided are important factors to attract patients to the HC

In China, the large scale health service institutions often have sufficient financial resources to renew the facilities and equipment and have comprehensive specialised departments. Thus, patients might prefer the large health service institutions when seeking health services. The standards of their judgements about good health service institutions include the scale, equipments, and types of health services provided by the health service institution, besides the qualification of health personnel and characteristics of the HC [1]. Therefore, the scale of the institution, medical equipment used and mix of health services provided are important factors to attract patients to the HCs.

### It is important for the HCs to have HIC

The coverage of present city health insurance is limited and it mainly compensates the third-tiered health service institutions. Thus, although many community health service institutions are contracted by the health insurance, the health service institutions covered by HIC are not many and the HIC rate to health service institutions is low [6,7]. But it is one of financial flows for the Chinese HCs. Therefore, it is important for the HCs to have HIC to increase the OW at the HCs.

### The women in childbearing age can influence the OW of the HCs

Very often women need more health services [26]. The results of our study also confirmed this issue. Especially in the community, the number of women in childbearing age can influence the OW at the HCs. Attracting women to HCs could be an important consideration when trying to increase the OW at the HCs.

## Conclusions

This paper aimed to identify the main factors that influence the increase of OW at Chinese HCs, and to provide strategies for the adjustment of national health policy in developing the community health service. The analysis suggested several key factors for increasing the OW: establishing the referral among the different levels of institutions; enhancing the qualification of health personnel and increasing the compensation of health insurance for services provided at HCs. Other key factors with a positive effect on the OW included: the government ownership of the HCs, the scale of the institutions, the medical equipment used, the mix of health services provided, and the women in childbearing age in the residence. Thus, in order to guide the patient to seek treatment in the primary HCs, the implementation of a 'gate-keeper' and an adequate referral process is needed, as well as improvements in the qualification of the HC health personnel and an increase in the HIC of the HC services.

## Abbreviations

HC: health centre; HS: health station; OW: outpatient workload; HOWG: high outpatient workload group; LOWG: low outpatient workload group; HI: health insurance; HIC: health insurance compensation.

## Competing interests

The authors declare that they have no competing interests.

## Authors' contributions

The main ideas for this analysis were proposed by JX, who was also responsible for writing the paper; YL, WW and JZ provided the data and revised the paper; MP provided suggestions for improvements; PY and HL were responsible for the database and all statistical analysis of data for this paper; ZL supervised the research process. The authors have all read the final version and agreed to publish in BMC Health Service Research.

## Authors' information

JX (1969, 7), lecturer, PhD student at the Department of Social Medicine and Health Management, School of Public Health, Tongji Medical College, Huazhong University of Science and Technology. Specialty is Health Service Research, and research on Chinese community health care management and policy. The latest published papers include " The Former Yugoslav Republic of Macedonia Health System Reform Summarization", *Chinese Journal of Social Medicine *, 2009, 26(1): 25-27; "The Analysis of Inpatient Bed Allocation Equity and Utilization in the City Community health service Center of China". *J Huazhong Univ Sci Technol *[ *Med Sci *], 2010, 30(2):141-144; "Analysis of medical insurance reimbursement rates of community health stations sponsored by various entities". *Chinese Journal of Hospital Administration*, 2010, 26(3):205-208, and "The Survey Analysis of the Health insurance Compensation Impact on the Income of City Community Health Center in 28 Chinese Cities", *Chinese Health Economics*, 2010,29(4):47-49. etc.

WW (1972,11), lecturer at the Jiangsu University, PhD student at the Department of Social Medicine and Health Management, School of Public Health, Tongji Medical College of Huazhong University of Science and Technology and researches in Health Service and Management. The latest published papers include " The study of outpatient health staff allocation efficiency of Shenzhen Maternal and Child Care Hopital" and "The study of inpatient health staff allocation efficiency of Shenzhen City Maternal and Child Care Hospital " in *Chinese Journal of Maternal and Child Care*, 2010, 25(8):1022-1023 and 25(9):1169-1170; "The Ukraine Health System Reform Summarization in *Chinese Journal of Social Medicine *in 2009, 26 ( 2 ):82-84; The influence of supporting life environments on the psychological health of the rural five-guarantee aged farmers in *Chinese Public Health Care *in 2006, etc.

YL (1981,11) , PhD student at the Department of Social Medicine and Health Management, School of Public Health, Tongji Medical College of Huazhong University of Science and Technology. Specialty is Health Service Research, and research in Chinese community health service management and policy. The latest published papers include "Slovakia Health System Reform Summarization" in *Chinese Journal of Social Medicine, 2008, 25(2): 80-82. and *"The Development, Effect and Concerned Issues of Pilot Work of National Community Health Service System Building in Key Contact Cities--Based on the Comprehensive Analysis of the Baseline Survey and Routine Monitoring Data" in *Chinese Journal of Social Medicine, 2009,26 (6):321-325*.

JZ (1980, 12), PhD student at the Department of Social Medicine and Health Management, School of Public Health, Tongji Medical College, Huazhong University of Science and Technology. Specialty is Health Service Research, and research in Chinese community health service management and policy. "Progress of medical insurance in community health facilities of the key contact cities" in Journal of Chinese Health Policy in 2010, "The comparison analysis of evaluation methods of scientific paper--study case of first-rate world biomedical scientists" in *Journal of Science Discipline Research*; "The frequency distribution analysis of order rank of scientific paper of first-rate world biomedical scientists--study case of Nobel prize winner of physiology or Medicine" in *Journal of Science and Technology Philosophical Research *in 2009.

MP (1971,9), Assistant professor (Health Economics) at the Department of Health Organisation, Policy and Economics, Faculty of Health, Medicine and Life Sciences, CAPHRI, Maastricht University Medical Center, Maastricht University, The Netherlands. Research areas: health care funding and management, out-of-pocket payments, stated preference methods in policy evaluation. Numerous publications in these research areas.

HL (1982, 10), MD, the doctor at Wuhan City Maternal and Child Health Care Centre. He has participated in 8 projects, such as "The study on the children's physical growth under 7 in the ten provinces in the rural China", "The study on the baby anaemia in three counties of Hainan Province", etc., and accumulated rich experience in the data collection and data analysis, and published some papers on the maternal and child health in the Chinese journals, such as Chinese Child Health Care, Chinese Maternal and Child Health Care, and Behaviour Medicine, etc.

PY ( 1965,8 ), PhD, assistant professor, the deputy director of Department of Epidemiology and Health Statistics, School of Public Health, Tongji Medical College, Huazhong University of Science & Technology; the research on biological statistical analysis and its application, and the evaluation of population health promotion performance. He has conducted more than 7 national and provincial projects and published 12 medical college textbooks and more than 20 papers in his field.

ZL (1959.9), Professor, PhD supervisor and the director of the Department of Social Medicine and Health Management, School of Public Health, Tongji Medical College, Huazhong University of Science and Technology, the famous national health expert and research on Chinese city community health service management and policy for more than two decades. He has published more than 78 papers on community health service research, and conducted 16 projects from the National Natural Science Foundation, Ministry of Health and Hubei Provinces, and wrote 7 books on health service.

## Pre-publication history

The pre-publication history for this paper can be accessed here:

http://www.biomedcentral.com/1472-6963/10/151/prepub
